# Observations on the Words, in Various Languages, Answering to the English Word Liver

**Published:** 1811-10

**Authors:** 


### *Observations on the Words, in various Languages, answering to the English Word Liver.*

(Month. Mag.)

SCAPULA, in his Greek Lexicon, observes, that "Λευχηπαλίας olim dicebatur *timidus*. Ajunt quorundam hepati vitium quoddam accidere, quod *eos timidos reddat;* ejus autem indicium vitiati Pallor est, qui tales timidos arguit."* From the Greek λευκηπαλίας, comes our vulgar phrase *white-liver'd*, an epithet frequently applied to cowardly and malicious characters.

In Italy the word *fegatoso* is applied to a person " *che*

* The author refers his readers to *Erasmi Chil.* Quære, What is the exact meaning of the Greek verb ηπαλίζω?

*ha*

*ha nella faccia del ribollimento, con pustule rosse preveniente da soverchio calore di sangue.*"

It may be further remarked, that our word *jealousy** seems to have been derived from *giallo*, on account of the yellowness of the skin of persons being tormented with this passion: so gloomy and uncomfortable views of any subject are commonly said to be taken with the *jaundiced eye*. In disordered states of the digestive organs, the secretions are sometimes so vitiated as to be changed in colour and consistency; the bile in particular often assumes a green appearance; the absorption of such bile would give the cornea of the eye a greenish cast; hence jealousy has been said to be a *green-eyed monster*.

The idea that was entertained of the great importance of this organ in the animal economy, may indeed be deduced from the etymology of the word itself. Our English word *liver* is derived from the Anglo-Saxon Lyfer, which comes from their verb Lyfian, *to live*. I shall be much obliged to any of your ingenious correspondents who may be able to trace the etymology of the words used to denote this organ in other languages; I have subjoined a list of a great many of them.

| | |
|---|---|
| Anglo-Saxon, | Lýfer. |
| English, | Liver. |
| German, | Leber. |
| Islandic, | Lifur. |
| Danish, | Lever. |
| Belgic, | Lever. |
| Dutch, | Lever. |
| Greek, | Ἧπαρ. |
| Latin, | Jecur. |
| Italian, | Fègato. |
| French, | Foie. |
| Spanish, | Higado. |

F.

* This word, however, has been by some etymologists derived from the Greek ζηλος.

